# Enhanced Performance of Sn‐Based Perovskite Photodetectors Through Double‐Sided Passivation for Near‐Infrared Applications

**DOI:** 10.1002/smll.202409592

**Published:** 2024-12-18

**Authors:** Yu Hsuan Lai, Chien Cheng Li, Yu Chuan Huang, Tzu Yu Huang, Xin Kai Gao, Chung Chi Yang, Chih Shan Tan

**Affiliations:** ^1^ Institute of Electronics National Yang Ming Chiao Tung University Hsinchu 30010 Taiwan

**Keywords:** near‐infrared, passivation, perovskite, photodetector, Sn‐based

## Abstract

The development of high‐performance Sn‐based perovskite photodetectors is presented with double‐sided passivation using large alkylammonium interlayers of PEAI and BDAI₂. This dual passivation strategy, applied to the top and bottom of FASnI₃ films, effectively improves film quality by reducing defect density, enhancing carrier mobility, and minimizing non‐radiative energy losses at the interfaces. At 720 nm, the photodetectors demonstrate a responsivity of 0.37 A W^−1^, a detectivity of 6.12 × 10¹^3^ Jones, and an external quantum efficiency (EQE) of 65.60%, with a rapid response time of 9 µs. Additionally, at 850 nm, the detectivity reaches as high as 3.27 × 10¹^3^ Jones. Furthermore, the device demonstrated a low 1/f noise of 1.21 × 10⁻¹⁵ AHz⁻⁰.⁵ at 10 Hz. Transient photocurrent (TPC) and transient photovoltage (TPV) measurements revealed a significant increase in charge recombination lifetime (τ_e_) and improved charge transfer efficiency. These results showcase the potential of Sn perovskite photodetectors for near‐infrared applications, including autonomous vehicles, biometric recognition, and biomedical treatments.

## Graphical abstract

At 720 nm, the photodetectors exhibit outstanding performance, with a responsivity of 0.37 A W^−1^, a detectivity of 6.12 × 10¹^3^ Jones, and an external quantum efficiency (EQE) of 65.60%, along with a fast response time of just 9 µs. At 850 nm, the detectivity reaches an impressive 3.27 × 10¹^3^ Jones.

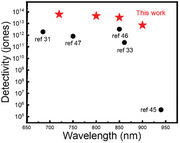

## 1 Introduction

Near‐infrared (NIR) photodetectors are specifically designed to absorb light in the near‐infrared spectrum (700–2500 nm) and convert it into electrical signals. Compared to visible light, NIR light features longer wavelengths and lower energy, which results in several advantages: reduced damage to biological samples, deeper tissue penetration, and minimal background autofluorescence interference in live biological systems.^[^ ^1^, ^2^, ^3^, ^4^, ^5^, ^6^, ^7^ ^]^ These characteristics make NIR photodetectors a powerful tool in medical diagnostics, surgery, and therapeutic applications. Beyond the medical field, NIR photodetectors are widely used in the pharmaceutical industry, environmental monitoring, and the oil and gas sectors, garnering significant attention over the past few decades.

Despite the notable success of semiconductor materials such as gallium arsenide, silicon, lead selenide, indium gallium arsenide, and other III‐V compound semiconductors in achieving high‐performance NIR photodetectors,^[^ ^8^, ^9^, ^10^, ^11^ ^]^ these materials present challenges. The strong covalent bonds in these inorganic semiconductors necessitate substantial energy for device operation. Moreover, traditional photodetector fabrication techniques—such as alloying, doping, etching, and epitaxy—are complex, expensive, and time‐consuming.

In recent years, there has been significant interest in the development of organic materials for optoelectronic applications.^[^ ^12^ ^]^ Among these, lead halide perovskites have emerged as promising semiconductor candidates, offering a unique combination of advantageous properties, including a low‐cost and scalable solution‐based fabrication process, high optical absorption coefficients,^[^ ^13^ ^]^ low trap state densities, and high carrier mobilities. These features give lead halide perovskites a competitive edge. While lead‐based perovskite photodetectors (PDs) have achieved impressive performance through compositional tuning, structural innovations, and the incorporation of buffer layers, these improvements often come at the cost of increased processing complexity and higher production expenses. Moreover, the wide bandgap of lead‐based perovskites (>1.5 eV, corresponding to a wavelength of 827 nm),^[^ ^14^, ^15^ ^]^ while effective in the visible spectrum, limits their absorption in the near‐infrared range. This results in a narrower response wavelength compared to commercial silicon‐based detectors, which can extend their response up to 1100 nm.^[^ ^16^, ^17^ ^]^ Additionally, the instability and toxicity associated with lead halides pose significant hurdles to the commercialization of perovskite‐based photodetectors.

Given these limitations, the development of non‐toxic, lead‐free perovskites has become a critical focus (including those based on Bi, Sn, Mn, Sb, Cu, and Yb),^[^ ^18^, ^19^, ^20^ ^]^ prompting a shift in research toward low‐lead or lead‐free alternatives. Among these, tin (Sn) stands out as the most promising candidate. As a Group IV element, Sn offers significantly lower toxicity compared to lead, while causing minimal disruption to the perovskite lattice. Sn^2^⁺ closely resembles Pb^2^⁺ in both valence state and ionic radius (Sn^2^⁺: 1.35 Å vs Pb^2^⁺: 1.49 Å), and it provides several key advantages, including a narrower bandgap (1.2–1.4 eV), lower exciton binding energy (≈18 meV), longer carrier diffusion lengths, and higher carrier mobility.^[^ ^21^, ^22^, ^23^, ^24^ ^]^ Most importantly, tin‐based perovskites extend the photodetection range into the near‐infrared region, making them a compelling alternative to lead‐based systems. As a result, extensive efforts have been devoted to optimizing lead‐free tin‐based perovskite photodetectors.

Research on pure tin‐based perovskite photodetectors remains limited, with current studies indicating that these materials generally underperform compared to lead halide perovskites, necessitating precise optimization for acceptable device performance. The primary challenge lies in the poor stability of tin‐based perovskites, which hampers further device development.^[^ ^25^, ^26^, ^27^ ^]^ Tin‐based detectors degrade rapidly in ambient conditions due to the susceptibility of Sn^2^⁺ to oxidation.^[^ ^28^ ^]^ This oxidation leads to the formation of tin vacancies within the perovskite lattice, inhibiting charge transport and negatively impacting device performance.

To address the oxidation issue, several strategies have been proposed, including compositional engineering,^[^ ^29^ ^]^ precursor engineering,^[^ ^27^ ^]^ and interfacial engineering.^[^ ^30^, ^31^ ^]^ Among these, the use of 2D layered perovskites has gained significant attention for improving the performance and stability of photodetectors. This approach involves replacing small MA⁺ or FA⁺ cations with larger organic cations, which significantly enhance stability. For instance, Miao He's team introduced phenylethyl ammonium (PEA⁺) additives into FASnI₃,^[^ ^32^ ^]^ which regulate film growth, passivate trap and defect states, inhibit Sn^2^⁺ oxidation, and alleviate crystal strain. The resulting FA₀.₈PEA₀.₂SnI₃ films exhibited high crystallinity and flexibility. Photodetectors utilizing FA₀.₈PEA₀.₂SnI₃ as the absorbing layer demonstrated exceptional performance, with a responsivity of 0.26 A W⁻¹ and a detectivity of 2.30 × 10¹¹ Jones. Furthermore, these devices exhibited rapid rise and decay times of 25 and 42 µs, respectively, outperforming state‐of‐the‐art Sn‐based perovskite detectors.^[^ ^32^ ^]^

Additionally, Yan Zhao's team proposed a double‐sided interface engineering strategy, in which a Sn‐Pb perovskite film is sandwiched between phenethylammonium iodide (PEAI) layers on both the top and bottom surfaces. The larger organic PEA⁺ cations align within the perovskite surface lattice, forming a 2D protective layer that shields the bulk perovskite from moisture and oxygen. Simultaneously, PEA⁺ passivates iodide anion defects at the bottom of the film. Compared to single‐sided passivation, Sn‐Pb mixed perovskite photodetectors with double‐sided passivation exhibited significantly improved performance and stability, including a broadband response from 300 to 1050 nm, a low dark current density of 1.25 × 10⁻^3^ mA cm⁻^2^ at −0.1 V, a fast response time of 35 ns, and stability exceeding 240 h. Moreover, the Sn‐Pb photodetector was integrated into an infrared conversion system, converting near‐infrared light into visible light. This double‐sided passivation approach presents a promising strategy for achieving high‐performance, stable perovskite photodetectors.^[^ ^33^ ^]^

In this work, we adopted a double‐interface passivation approach for the perovskite absorption layer, utilizing a large alkylammonium intermediate layer on both the top and bottom of the perovskite layer to reduce energy loss at the transport layer‐perovskite interface. The use of this large alkylammonium intermediate layer not only suppresses non‐radiative energy loss but also effectively guides the growth of the perovskite film, resulting in higher crystallinity. More importantly, the intermediate layer inhibits phase separation on the perovskite's top surface, ensuring a more uniform interface throughout the structure.^[^ ^34^ ^]^ We replaced the commonly used PEAI additive in the bottom passivation layer with BDAI₂. Previous reports^[^ ^3^, ^34^ ^]^ suggest that BDAI₂, compared to PEAI, promotes larger perovskite grain sizes and reduces trap density. This is likely because the BDAI₂ layer, with its inorganic [PbX₆]⁴⁻ sheets exposed in the Dion‐Jacobson (D‐J) phase, achieves better lattice matching with the upper 3D perovskite compared to the Ruddlesden‐Popper (R‐P) and alternating cation interlayer (ACI) phases. Our findings confirmed this phenomenon, as the combination of BDAI₂ and PEAI outperformed both the control group and the PEAI/PEAI combination in key performance metrics, including EQE, responsivity, noise current, and detectivity. Notably, the detectivity reached a record‐high value of 6.12 × 10¹^3^ Jones, the highest reported for pure tin‐based perovskite photodetectors to date.

## 2 Results and Discussion

**Figure 1:**
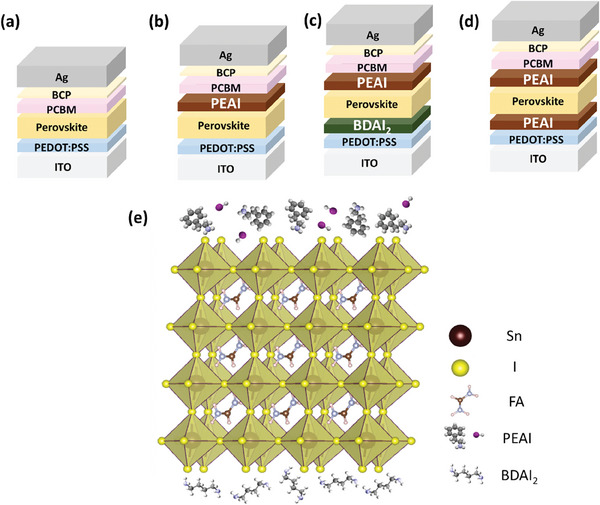
Schematic Representation of Device Architecture and Passivation Mechanism. a–d) Illustrations of photodetector device structures incorporating various combinations of passivated interfaces. e) Detailed schematic depicting the interaction and passivation mechanism between the passivated interfaces and the Sn‐based perovskite layer.

As shown in **Figure** **1**a–d, we tested four different interface passivation combinations. Figure 1a represents the control group without any additives, while Figure 1b introduces a PEAI layer on top of the perovskite. In Figure 1c,d, an additional layer of BDAI₂ and PEAI, respectively, was applied beneath the perovskite layer, allowing us to study the effects of each intermediate layer and interface passivation separately. Figure S1 (Supporting Information) illustrates the chemical structures of PEAI and BDAI₂. These compounds were dissolved in a mixed DMF and DMSO solvent and spin‐coated onto the PEDOT layer, followed by deposition of the FASnI₃ perovskite layer using a two‐step spin‐coating process. During this process, the large alkylammonium interlayer can interact with the perovskite precursor in the DMF/DMSO solvent mixture. Given the high concentration of alkylammonium ions at the interface, these ions likely incorporate into the interlayer between the PEDOT and the upper 3D perovskite, as depicted in Figure 1e. In the second spin‐coating step, PEAI cations were introduced into the antisolvent at a concentration of 0.5 m, and the layer was completed by thermal annealing.

**Figure 2:**
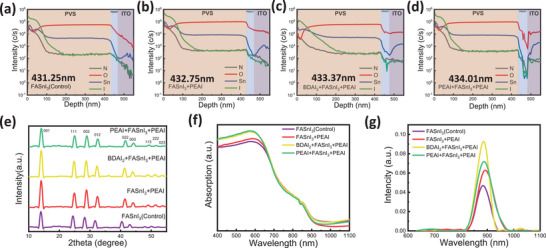
Characterization of Perovskite Films: SIMS, XRD Patterns, UV–vis Absorption, and PL Spectra. a–d) SIMS profiles for the ITO/PEDOT/perovskite layers. e) XRD patterns of the control film compared to perovskite films fabricated with various combinations. f) UV–vis absorption spectra of the control film and perovskite films with different combinations, all prepared on glass substrates. g) Steady‐state PL spectra of the control film and perovskite films with different combinations, all prepared on glass substrates.

To confirm the formation of a separate 2D perovskite layer, a series of material characterization techniques were employed across all four devices. As shown in **Figure** **2**a–d, Secondary Ion Mass Spectrometry (SIMS) was used to determine the thickness of the devices. The intermediate layer was not distinctly detected; however, a slight increase in the overall perovskite thickness of about 2–3 nm was observed. Figure 2e presents the X‐ray diffraction (XRD) patterns of the perovskite films, which were analyzed to study the crystal structure. Despite the inclusion of different intermediate layers, all the perovskite films exhibited similar characteristic XRD patterns. But molecular doping in perovskite materials causes a shift of X‐ray diffraction (XRD) peaks, such as (111), (002), (012), (022), and (003), to higher angles, primarily due to lattice contraction. When BDA^2^⁺ and PEA⁺ molecules are incorporated into the FASnI₃ crystal structure, they can reduce interatomic distances if the dopant ions are smaller or if they introduce compressive strain, both of which decrease the interplanar spacing. Similarly, UV–vis absorption measurements, shown in Figure 2f, did not provide any evidence of 2D perovskite formation. To further verify the existence of the intermediate layer on PEDOT, X‐ray photoelectron spectroscopy (XPS) measurements were conducted on both PEDOT and modified PEDOT. The results confirmed the presence of the intermediate layer, as indicated by the additional I 3d doublet peaks at 618 and 631 eV (Figure S2, Supporting Information). This confirms that, after mixing PEAI and BDAI₂ cations with DMF and DMSO and spin‐coating them onto the hole transport layer, a certain amount of PEAI cations remained on the PEDOT surface (**Table** **1**).^[^ ^35^ ^]^

**Table 1:** Performance Characterization of Sn‐Based Perovskite Films.

	FASnI3 [Control]	FASnI3+PEAI	BDAI_2_+FASnI3+PEAI	PEAI+FASnI3+PEAI
C(F)	1.22 × 10^−9^	2.69 × 10^−10^	1.12 × 10^−10^	2.01 × 10^−10^
d(nm)	431.25	432.75	433.37	434.01
ɛ_*r*_	14.86	3.29	1.37	2.46
*n_trap_* (cm^−3^)	7.14 × 10^14^	1.44 × 10^14^	5.65 × 10^13^	1.07 × 10^14^
µ(cm^2^ V⁻¹s⁻¹)	0.02	0.10	0.32	0.15
Lifetime(ns)	0.69	1.55	2.78	2.10

Next, ultraviolet‐visible (UV‐Vis) absorption spectroscopy was employed to evaluate the light‐harvesting capability of the perovskite and assess whether the PEAI and BDAI₂ protective layers impacted the optoelectronic properties of the perovskite (Figure 2f). The results showed that all spectra exhibited similar profiles and absorption edges, indicating that the intermediate layers had minimal effect on the absorption and bandgap of the perovskite films. Subsequently, steady‐state photoluminescence (PL) measurements were performed (Figure 2g). The PL intensity of the sample with a PEAI layer on top of the perovskite was ≈1.5 times higher than that of the control group. Notably, samples with protective layers on both sides of the perovskite film showed even greater enhancements, with the PEAI+PEAI combination exhibiting a 1.75‐fold increase and the BDAI₂+PEAI combination achieving a 2.25‐fold increase compared to the control group. These results strongly suggest that non‐radiative recombination was significantly suppressed by the intermediate layers^[^ ^34^ ^]^ The redshift of the PL peak in Figure 2g is likely caused by BDA^2^⁺ and PEA⁺ doping in the perovskite material, which introduces lattice strain, just like the XRD results. When BDA^2^⁺ and PEA⁺ are incorporated into the crystal structure, they create compressive strain, as shown in Figure 2e, altering interatomic distances and modifying the band structure. This strain often reduces the bandgap, lowering the emission energy and shifting the PL peak to a higher wavelength. Additionally, the small photoluminescence (PL) peak beyond 1000 nm is due to the formation of new energy levels induced by BDA^2^⁺ and PEA⁺ doping, often indicating carrier recombination associated with dopant‐induced strain (**Table** **2**).

**Table 2:** Performance Characterization of Sn‐Based Perovskite Photodetectors.

	FASnI_3_ [Control]	FASnI_3_+PEAI	BDAI_2_+FASnI_3_+PEAI	PEAI+FASnI_3_+PEAI
EQE (%)	58.47	63.31	65.60	63.46
Responsivity (A/W)	0.33	0.36	0.37	0.36
Noise current (A/(Hz)^0.5^)	1.23 × 10^−12^	5.92 × 10^−13^	1.21 × 10^−15^	2.22 × 10^−14^
Detectivity (jones)	5.31 × 10^10^	1.20 × 10^11^	6.12 × 10^13^	3.24 × 10^12^

**Figure 3:**
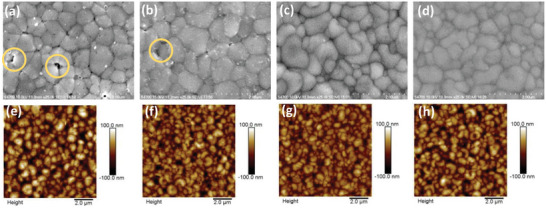
Impact of Passivated Interface Combinations on Mixed Perovskite Films. a–d) Top‐view SEM images of perovskite films: a) Control, b) FASnI₃+PEAI, c) BDAI_2_+FASnI₃+PEAI, and d) PEAI+FASnI₃+PEAI. e–h) Corresponding AFM images for each respective perovskite film.

Surface SEM images (**Figure** **3**a–d)) also did not show signs of a distinct surface 2D perovskite layer. These findings, combined with the minimal amount of PEAI introduced during the antisolvent step, indicate that the intermediate layer does not significantly affect the perovskite crystallization process.^[^ ^35^ ^]^ Furthermore, FASnI₃ films with different passivation combinations were characterized using scanning electron microscopy (SEM) and atomic force microscopy (AFM), as shown in Figure 3a–h. In the control sample without any additives (Figure 3e), the perovskite film exhibited uncontrolled crystallization, leading to poor uniformity and inadequate interconnections. The AFM images revealed significant surface roughness, with voids as deep as 37 nm and noticeable pinholes, which could contribute to current leakage in the photodetectors. However, after introducing protective layers, the perovskite films displayed a much smoother, denser morphology, with better uniformity and larger grain sizes. This was particularly evident in the perovskite films with double‐sided protection, where surface pinholes were significantly reduced or eliminated (Figure 3c,d). This improvement in film quality is crucial for efficient charge transport, suggesting that the BDAI₂ and PEAI additives effectively optimize the film by influencing the preferential growth direction of the perovskite grains.

**Figure 4:**
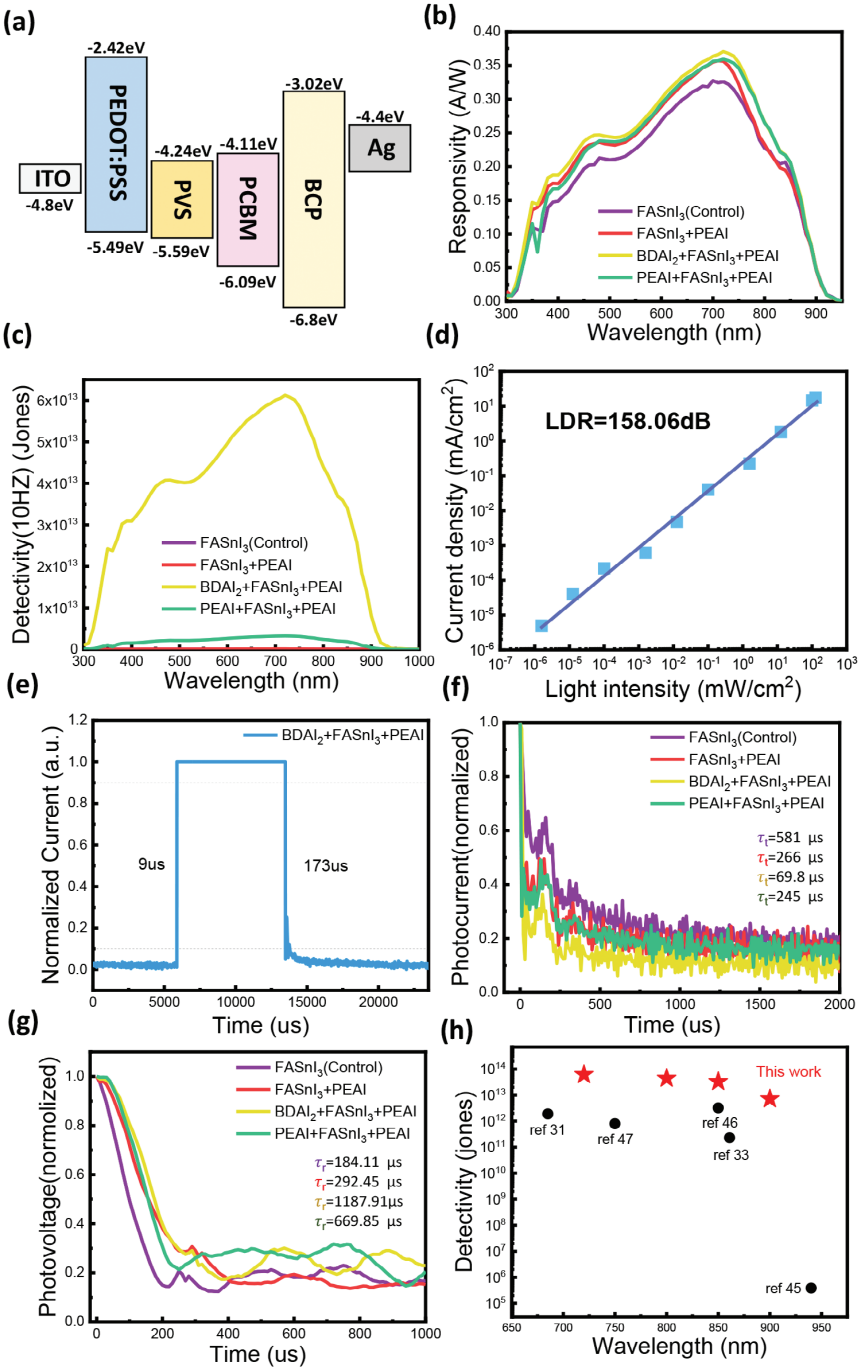
Performance Characterization of the Sn‐Based Perovskite Photodetector. a) The energy band alignment of the device. b) Wavelength‐dependent responsivity curve. c) Wavelength‐dependent detectivity curves are calculated by EQE and noise current under 10Hz. d) Light intensity‐dependent photocurrent of the device with BDAI_2_+FASnI_3_+PEAI. e) Rising and falling edges for determining the response time of the device with BDAI_2_+FASnI_3_+PEAI. f,g) Normalized transient photovoltage (TPV) and normalized transient photocurrent (TPC) spectra of control and passivated devices. h) Comparison of the critical parameters of Sn‐Pb perovskite PD.^[^ ^44^, ^45^, ^46^ ^]^

These observations are supported by the AFM results shown in Figure 3e–h. The root‐mean‐square (RMS) roughness for the control group, PEAI top layer, BDAI₂+PEAI, and PEAI double‐layer films were 37.0, 30.3, 24.8, and 32.3 nm, respectively, indicating that the BDAI₂ + PEAI combination produced the smoothest surface. A smoother surface enhances the on/off current ratio in near‐infrared (NIR) photodetectors based on FASnI₃ films.^[^ ^32^ ^]^ The SEM analysis further confirms that the crystallization kinetics of FASnI₃ films can be modulated by the addition of PEAI and BDAI₂, improving surface morphology. The fabrication process of tin‐based perovskite photodetectors with a layered structure is illustrated in Figure 1a–d. Figure 4a shows the energy levels of the materials used in the device, calculated from UPS and UV–vis absorption spectra (Figure S3, Supporting Information). Upon light irradiation, electrons excited in the Sn‐based perovskite layer are transported through the PCBM electron transport layer to the Ag electrode, while holes travel through the PEDOT hole transport layer to the ITO electrode. This charge separation generates a current, which is measured. Since the current is directly proportional to the intensity of the incident light, this method allows for accurate measurement of both the energy and intensity of the light.^[^ ^36^ ^]^

The external quantum efficiency (EQE) illustrates the photodetector's capability to convert incident photons into charge carriers across different wavelengths. As shown in Figure S4 (Supporting Information), the tin‐based photodetector demonstrates a broad visible‐near‐infrared response spectrum, spanning from 300 to 900 nm. Notably, after the introduction of additives, there is a significant overall enhancement in EQE, particularly with the BDAI₂ + PEAI combination. The EQE increased from 58% in the control group to 65%, representing a 7% improvement, and maintained this value in the near‐infrared (NIR) region. This is the highest EQE reported so far for pure tin‐based photodetectors.^[^ ^32^ ^]^ This improvement can be attributed to the low bandgap of 1.34 eV in Sn‐based materials, as well as the effective passivation of defects within the perovskite layer. The high EQE in the NIR region also contributes to improved responsivity (R), which is a key parameter for evaluating the photodetector's performance. Generally, responsivity is closely related to EQE and can be calculated according to Equation (1).

(1) $$R=\frac{EQE\times e}{h\upsilon }\;$$

The charge of an electron is denoted as e, Planck's constant as h, and the frequency of the light signal as υ. The responsivity, calculated from the EQE curve, is presented in **Figure** **4**b. The results show that in the near‐infrared range, the BDAI₂ + PEAI combination achieves an optimal responsivity of 0.37 A W^−1^ at ≈720 nm.

Noise is a key factor limiting the sensitivity of photodetectors. It refers to random fluctuations around the signal's mean value and can be classified into three types: shot noise, thermal noise, and 1/f noise. While 1/f noise is frequency‐dependent, shot and thermal noise are not. The total noise current of the hybrid Sn‐based photodetector was measured at zero bias using a fast Fourier transform (FFT) of the dark current as a function of time, as shown in Figure S5 (Supporting Information). At low frequencies, 1/f noise dominates the noise current, which can be mitigated by operating the device at higher frequencies. This type of noise originates mainly from local electronic state fluctuations due to disorders or defects. The BDAI₂ + PEAI combination exhibits a significant reduction in 1/f noise, decreasing by nearly three orders of magnitude compared to the control group. This improvement is attributed to the more uniform, well‐oriented film with fewer defects, demonstrating that effective defect passivation can substantially enhance photodetector performance.^[^ ^29^ ^]^ In Figure S5 (Supporting Information), the noise current curve of BDA^2^⁺ and PEA⁺ doping shows a section where the noise current increases with frequency, attributed to the influence of trap states and charge carrier dynamics. As illustrated in Figure 2g, BDA^2^⁺, and PEA⁺ doping introduce additional energy levels that act as trapping sites for charge carriers. At lower frequencies, these trapped carriers can respond effectively to the external electric field, resulting in relatively stable noise characteristics. However, as the frequency increases, the trapped carriers are less able to follow the alternating field efficiently, causing increased fluctuations in the charge distribution.

The detectivity (D^*^) in Figure S6 (Supporting Information) is a parameter used to evaluate sensitivity and noise resistance, representing the device's ability to detect weak light, making it another key parameter for high‐performance PDs. Previous studies have proposed two common methods for calculating D^*^. Some researchers^[^ ^37^, ^38^, ^39^ ^]^ used a simplified equation, which can be given as follows:

(2) $$D^{*}=\frac{R}{\sqrt{2qJ_{dark}}}\;$$

where R is the responsivity, *q* is the charge value of an electron, and *J_dark_* is the dark current density, as shown in Figure S7 (Supporting Information). However, this method accounts only for shot noise from the dark current while neglecting frequency‐dependent noise (1/f noise). To address this, some researchers^[^ ^29^, ^40^, ^41^, ^42^ ^]^ have adopted an alternative, commonly used algorithm that includes the influence of 1/f noise for more comprehensive noise analysis.

(3) $$D^{*}=\frac{\sqrt{A\Delta f}}{NEP}=\frac{R\sqrt{A\Delta f}}{i_{n}}\;$$

where A is the device area, ∆f is the bandwidth, and noise equivalent power (NEP) represents the minimum light intensity that the photodetector can distinguish from noise. Here, *i_n_* represents the total noise current, and R represents the responsivity. One significant challenge in achieving a high D* is reducing the dark current density (*J_dark_*) and noise current (*i_n_*). Therefore, effectively suppressing the dark current can significantly contribute to achieving a high D* in photodetectors (**Table** **3**).

**Table 3:** Comparison of the critical Parameters of Sn‐based NIR Perovskite Photodetectors. (Using noise current to calculate detectivity).

Perovskite	Structural	EQE [%]	D* [jones]	R [A/W]	Response speed	Noise current A/ [Hz]^0.5^	Refs.
CsSnI_3_	–	7.14%@940 nm	3.85 × 10^5^@940 nm	0.05@940 nm	84/243 ms	–	[44]
FA_0.8_PEA_0.2_SnI_3_	photodiode	37.81%@861 nm	2.3 × 10^11^@861 nm	0.262@861 nm	25/42 µ*s*	2.60 × 10^−13^	[32]
FASnI_3_	phototransistor	78%@685 nm	1.9 × 10^12^@685 nm	0.43@685 nm	180/360 s	1.50 × 10^−15^@1Hz	[30]
FASnI_3_	phototransistor	–	3.20 × 10^12^@850 nm	–	117/206 s	2.00 × 10^−9^@1Hz	[45]
PEA_0.85_FA_0.15_SnI_3_	photodiode	57.98%@750 nm	8.00 × 10^11^@750 nm	0.35@750 nm	0.78/– µ*s*	–	[46]
FASnI_3_	photodiode	65.60%@720 nm 30.11%@850 nm	6.12 × 10^13^@720 nm 3.27 × 10^13^@850 nm	0.37@720 nm 0.21@850 nm	9/173 µ*s*	1.20 × 10^−15^@10Hz	This work

Based on the noise current and responsivity R, the resulting detectivity (D^*^) curve is shown in Figure 4c. The maximum D^*^ values at 720 nm (in the near‐infrared region) for the reference device and devices passivated with PEAI, BDAI₂+PEAI, and PEAI+PEAI are ≈5.31 × 10¹⁰, 1.20 × 10¹¹, 6.12 × 10¹^3^, and 3.24 × 10¹^2^ cm·Hz^0.5^·W⁻¹, respectively. The device passivated with BDAI₂+PEAI exhibits a D^∗^ value 18.89 times higher than that of the PEAI+PEAI passivated device, and more than three orders of magnitude greater than the reference device. This represents a significant improvement, highlighting the potential of this combination to substantially enhance photodetector performance. We also found that this photodetector has a detectivity as high as 3.27× 10¹^3^ Jones at 850nm.

To further validate the weak light detection capabilities of our device, we evaluated its Linear Dynamic Range (LDR), a critical performance metric for photodetectors that reflects response stability across varying incident light intensities. For a high‐quality near‐infrared photodetector, an ideal LDR signifies a stable photoresponse over a wide range of light intensities, from very low to very high. As shown in Figure 4d, the BDAI₂+PEAI combination exhibits the broadest LDR, reaching an impressive value of 158.06 dB. Furthermore, the device can detect a minimum light intensity as low as 10⁻⁶ mW cm^−^^2^, demonstrating its exceptional capability for weak light detection.

Response speed, another critical parameter for photodetectors, represents the device's ability to track rapidly changing optical signals. In this experiment, the rise and fall times of the photocurrent were measured by rapidly switching the light source. The rise time is defined as the time required for the output signal to increase from 10% to 90% of its maximum value, while the fall time is defined as the time required for the signal to decrease from 90% to 10%.^[^ ^33^ ^]^ The BDAI₂ + PEAI passivation combination, which showed the best performance in previous tests, was selected for direct testing (Figure 4e). This passivation layer effectively passivated surface defects on the perovskite, reducing defect‐induced recombination in the active layer. As a result, the device exhibited an excellent rise time of 9 µs and a fall time of 173 µs.

The transient photocurrent (TPC) and transient photovoltage (TPV) measurements were conducted to analyze the transport mechanism of photogenerated charge carriers in the solar cells. From these measurements, the charge transfer lifetime (from TPC) and charge recombination lifetime (from TPV) were determined, providing insights into the internal structure and carrier dynamics of the device. The carrier transfer time (τ_t_) was obtained by fitting the TPC decay using the equation I(t) = I_0_ exp(‐t/τ_t_), where I_0_ represents the steady‐state photocurrent (Figure 4f). In this case, τ_t_ decreased from 581 µs in the control to 69.8 µs, indicating faster charge transfer. The more rapid TPC decay in the BDAI₂ + PEAI passivated device reflects higher charge transfer and extraction efficiency, which can be attributed to reduced defects and improved perovskite film quality.^[^ ^43^ ^]^ The carrier recombination lifetime (τ_e_) was derived by fitting the TPV decay using the equation V(t) = V_0_ exp(‐t/τ_e_), where V_0_ represents the steady‐state photovoltage (Figure 4g). In devices with both upper and lower protective layers, τ_e_ increased significantly from 184.11 µs in the control sample to 1187.91 µs, indicating that the passivation layers effectively reduced non‐radiative recombination at the perovskite/ETL interface.

To elucidate the double‐sided passivation mechanism, particularly in terms of charge recombination and defect density, time‐resolved photoluminescence (TRPL) measurements were conducted. TRPL is a powerful technique for probing the rapid carrier dynamics of optoelectronic materials, providing critical insights into carrier recombination rates, defect state density, and carrier lifetimes.^[^ ^29^ ^]^ After applying different passivation layer combinations, all exhibited improved carrier lifetimes compared to the control group, with the BDAI₂ + PEAI combination demonstrating the best performance in Figure S8 (Supporting Information). This combination extended the carrier lifetime by several nanoseconds, reaching a remarkable level. Such a significant improvement indicates that the double‐sided passivation method effectively reduces defect state density and enhances crystal quality, resulting in less non‐radiative recombination and allowing carriers to sustain longer lifetimes before recombination occurs. These findings are consistent with the observed improvements in device performance, including enhanced responsivity and reduced noise current.

Due to the absence of a relative dielectric constant (ɛ_*r*_) data, we first measured the frequency‐dependent capacitance curves to calculate the ɛ_*r*_ values for the different perovskite films, with the results presented in Figure S9 (Supporting Information). Using these values, we substituted them into the n_trap_ formula to calculate the hole trap density. The BDAI₂ + PEAI passivated film exhibited a hole trap density of ≈5.65 × 10¹^3^ cm⁻^3^, which is significantly lower than that of the control group (7.14 × 10¹⁴ cm⁻^3^), the PEAI passivated group (1.45 × 10¹⁴ cm⁻^3^), and the PEAI+PEAI passivated group (1.07 × 10¹⁴ cm⁻^3^). This result provides direct evidence that BDAI₂ + PEAI passivation more effectively reduces trap states in tin‐based perovskite films.

To further investigate the impact of passivation on hole mobility and hole trap density, single‐carrier devices with various passivation methods were fabricated and tested, as shown in Figures S10 and S11 (Supporting Information). To confirm the reduction in defect density, space‐charge‐limited current (SCLC) measurements were performed on hole‐only devices. The device structure was ITO/PEDOT/perovskite/MoO₃/Ag, and the formula for calculating hole trap density is as follows:

(4) $$n_{trap}=\frac{2\varepsilon_{0}\varepsilon_{r}V}{qd^{2}}\;$$

where ɛ_0_ is the vacuum dielectric constant, ɛ_*r*_ is the relative dielectric constant of the perovskite material, q is the electron charge, d is the perovskite film thickness, and V_TFL_ is the trap‐filled limit voltage.

On the other hand, the dark current follows the Mott‐Gurney law:

(5) $$J\;=\frac{9\varepsilon_{0}\varepsilon_{r}\mu V^{2}}{8d^{3}}$$

(6) $$\mathrm{slope}\;=\frac{\sqrt{J}}{V}=\sqrt{\frac{9\varepsilon_{0}\varepsilon_{r}\mu }{8d^{3}}}$$

where ɛ_0_ is the vacuum dielectric constant, ɛ_*r*_ is the relative dielectric constant of the perovskite material, and d is the perovskite film thickness. The carrier mobilities of the perovskite films for the control group, PEAI passivation, BDAI₂ + PEAI passivation, and PEAI+PEAI passivation were calculated to be 0.02, 0.10, 0.32, and 0.15 cm^2^ V⁻¹s⁻¹, respectively. These results demonstrate that BDAI₂ + PEAI passivation significantly enhances carrier mobility, increasing it by more than 14 times compared to the control group. Finally, the reduction of carrier recombination and defect density leads to more sensitive photodetection and faster response times, attributed to improved carrier mobility.

**Figure 5:**
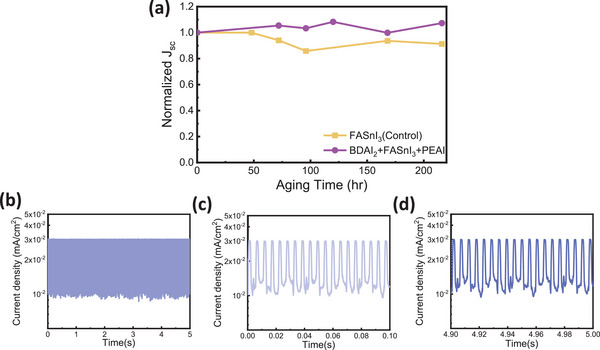
Stability test of photodetector. a) Stability curves of different passivation were stored in the glove box and tested under AM 1.5G solar illumination. b) The unencapsulated BDAI_2_+PEAI PD were tested for more than 1000 repeated on‐off stability measurements under the atmosphere. c) On‐off stability for the first 20 times. d) On‐off stability for the last 20 times.

The stability of photodetectors (PDs) is a critical factor for practical applications, encompassing both operating and shelf stability. To assess shelf stability, devices were stored in an N₂ glove box, and their performance was evaluated (**Figure** **5**a). Notably, the BDAI₂+PEAI devices exhibited excellent storage stability, retaining their performance even after 220 h of storage. In contrast, the control devices showed a decline, maintaining only 91.33% of their initial photoresponse over the same period. On‐off stability is another essential parameter, reflecting the device's ability to operate consistently over time. Unencapsulated PDs were tested for on‐off stability under atmospheric conditions (Figure 5b). Remarkably, the devices achieved rapid current switching, completing 1000 cycles within five s (at a switching rate of 40 cycles in 0.2 s). Throughout the test, the response current remained stable, as evidenced by the consistent performance during both the first 20 cycles (Figure 5c) and the last 20 cycles (Figure 5d) within the 1000 cycles in Figure 5b, showing no signs of attenuation.

## 3 Conclusion

In conclusion, we successfully developed high‐performance Sn‐based perovskite photodetectors using a double‐sided passivation strategy with large alkylammonium interlayers of PEAI and BDAI₂. This approach applied to the top and bottom of FASnI₃ films, led to improved film quality, characterized by uniform morphology, reduced defect density, and enhanced carrier mobility, while also minimizing non‐radiative energy losses at the interfaces. The dual passivation method significantly lowered dark current and noise density, achieving a remarkably low 1/f noise of 1.21 × 10⁻¹⁵ AHz⁻⁰.⁵ at 10 Hz. The optimized Sn‐based photodetector exhibited excellent performance, with a responsivity of 0.37 A W^−1^, a detectivity of 6.12 × 10¹^3^ Jones, a response time of 9 µs, and an EQE of 65.6%. These results demonstrate superior performance among pure Sn‐based detectors and underscore the potential of Sn perovskites for advanced near‐infrared photodetector applications in fields such as autonomous vehicles, biometric recognition, and biomedical treatments.

## 4 Experimental Section

### Materials

Tin(II) fluoride (SnF₂, 99.8%), chlorobenzene (CB, 99.8%), and bathocuproine (BCP, 99.5%) were sourced from Sigma‐Aldrich, while N,N‐dimethylformamide (DMF, 99.8%) was obtained from ACROS. Tin(II) iodide (SnI₂, 99.999%) was purchased from ALFA, and anhydrous dimethyl sulfoxide (DMSO, 99.8%) was supplied by UNI‐REAGEN. CH₃(NH₂)₂I (FAI, 99.5%), phenylethylammonium iodide (PEAI, 99.5%), and 1,4‐butanediammonium iodide (BDAI₂, 99.5%) were acquired from Greatcell Solar Materials.

### Preparation of Perovskite Precursor Solution and Upper and Lower 2D Protective Layers

The chemical formula of the perovskite solution used in this work was FASnI₃. To prepare a 0.65 m perovskite solution, FAI (111.78 mg), SnI₂ (242.13 mg), and SnF₂ (15.62 mg) were dissolved in a mixed solvent of DMSO and DMF in a 1:4 volume ratio. Both PEAI and BDAI₂ were dissolved in the same DMSO/DMF solvent mixture to create 3 mg mL⁻¹ solutions for use as the lower protective layer of the perovskite. For the upper protective layer, PEAI was dissolved in chlorobenzene to form a 0.5 mg mL⁻¹ solution, which was stirred overnight at room temperature.

### Fabrication of the Perovskite Photodetector

The glass substrates with ITO were ultrasonically cleaned using acetone and deionized water, followed by UV‐ozone treatment. A PEDOT:PSS layer was then spin‐coated onto the substrates at 4000 RPM for 30 s and thermally annealed at 150 °C for 15 min in ambient conditions. All subsequent steps in preparing the tin‐based perovskite films were carried out inside a glovebox. First, 60 µL of the PEAI or BDAI₂ solution was spin‐coated onto the PEDOT:PSS layer at 5000 RPM for 30 s without annealing. The perovskite precursor was then spin‐coated using a two‐step process: 1000 RPM for 30 s, followed by 5000 RPM for 30 s, with 300 µL of anti‐solvent chlorobenzene added at the 15 s of the second step. The resulting film was thermally annealed at 100 °C for 10 min.

Next, PCBM and BCP were sequentially spin‐coated onto the perovskite layer. For PCBM (20 mg mL⁻¹ in chlorobenzene), 50 µL was spin‐coated at 1000 RPM for 20 s, followed by annealing at 100 °C for 3 min. BCP (0.5 mg mL⁻¹ in isopropanol) was spin‐coated at 5000 RPM for 15 s without annealing. Finally, a 100 nm thick Ag electrode was thermally evaporated to complete the photodetector fabrication, yielding a device area of 0.04 cm^2^. The absorption spectra of the films were measured using a UV–vis spectrophotometer (Hitachi U‐4100), and XRD patterns were obtained with an Ital Model IS2773‐00LFF (40KV, 50 mA) to analyze the crystal structure of the perovskite. Photoluminescence (PL) spectra were recorded using an LQ‐100X measuring system from Enlitech Co. Ltd., equipped with a 405 nm excitation laser and a 100 mm integrating sphere. SEM images were captured using a high‐resolution field emission scanning electron microscope with an energy‐dispersive spectrometer (Hitachi S‐4700I). The 2D surface morphology was analyzed using a Bruker Dimension Icon Scanning Probe Microscope (ICON) with a 10 × 10 µm planar scan range.

*J*–*V* curves (dark current) were recorded using a Keysight B1500A analyzer, and TRPL curves were plotted using a PicoHarp300 in TTTR Mode with a PHR 800 router (PicoQuant). XPS and UPS spectra were collected using a Thermo Fisher Scientific ESCALAB Xi+ system with a He I ultraviolet radiation source (21.22 eV), and the energy scale was calibrated to the Fermi edge of gold (0 eV). EQE data were acquired using the Solar Cell Quantum Efficiency Measurement System (QE3000) with a Si detector (300–1100 nm). Noise current curves were measured using a ProPlus 9812B wafer‐level 1/f noise characterization system. Response times (current‐time) were measured using a Keysight B1500A analyzer, coupled with a custom‐built high‐speed fan and an AM1.5G simulated solar light spectrum. Capacitance‐frequency measurements were performed using an Agilent 4284A Precision LCR Meter. All measurements were conducted in a nitrogen‐filled glovebox, and the devices were left unencapsulated.

### Associated Content

Additional calculation results (PDF).

## Conflict of Interest

The authors declare no conflict of interest.

## Author Contributions

Y.H.L. conducted the perovskite thin film fabrication, performed the measurements, and authored the paper. C.C.L., Y.C.H., and X.K.G. provided experimental assistance. T.Y.H. contributed to the manuscript refinement. C.C.Y. assisted with drawing the structural diagrams. C.S.T. provided supervision, guidance, and further refinement of the manuscript.

## Supporting information



Supporting Information

## Data Availability

The data that support the findings of this study are available from the corresponding author upon reasonable request.
