# Supplementary material for: Enhanced Performance of Sn‐Based Perovskite Photodetectors Through Double‐Sided Passivation for Near‐Infrared Applications
Source: Small. 2024 Dec 18;21(5):2409592. doi: 10.1002/smll.202409592 (PMC11798353; doi:10.1002/smll.202409592)
Supplement: Supplementary file 1 — Supporting Information [file SMLL-21-2409592-s001.docx]

**Enhanced Performance of Sn-Based Perovskite Photodetectors Through Double-Sided Passivation for Near-Infrared Applications**

Yu Hsuan Lai^1^, Chien Cheng Li^1^, Yu Chuan Huang^1^, Tzu Yu Huang^1^, Xin Kai Gao^1^, Chung Chi Yang^1^, Chih Shan Tan^1*^

^1^ Institute of Electronics, National Yang Ming Chiao Tung University, Hsinchu 30010, Taiwan

**Figure S1. Chemical Structures of PEAI and BDAI Cations.**

**Fig. S2. X-ray photoelectron spectroscope (XPS)**

I 3d spectra measured on (a) ITO/PEDOT: PSS, (b) ITO/PEDOT: PSS/BDAI, (c) ITO/PEDOT: PSS/PEAI. ****

**Figure. S3. UPS and UV-Vis Absorption to calculate bandgap and EVB.**

(a)UPS measurements can be used to confirm the highest price band energy of a material. (b)Convert the absorption spectrum to a Tauc plot to obtain the energy gap width of the material

|  | $\boldsymbol{\Phi=hv-}\boldsymbol{E}_{\boldsymbol{bMAX}}$ | (S1) |
| --- | --- | --- |
|  | $\boldsymbol{E}_{\boldsymbol{VB}}\boldsymbol{=\Phi-}\boldsymbol{E}_{\boldsymbol{bmin}}$ | (S2) |
|  | $\boldsymbol{Ahv}^{\boldsymbol{0.5}}\boldsymbol{=C\times(hv-}\boldsymbol{E}_{\boldsymbol{g}}\boldsymbol{)}$ | (S3) |

Equations S1 and S2 are the conversion formulas for $\boldsymbol{E}_{\boldsymbol{VB}}$ ​, where 𝚽 is Planck's constant, 𝒗 is the optical frequency, and the measurement setup uses HeI as the excitation source (hν=21.2eV). $\boldsymbol{E}_{\boldsymbol{bMAX}}$ represents the cut-off energy, while $\boldsymbol{E}_{\boldsymbol{bmin}}$ denotes the highest electron-filling energy. Equation S3 is the bandgap calculation formula, where A is the material's light absorption intensity, and C is a general constant.

**Figure S4. The EQE spectra of the Sn-Based Perovskite Photodetector.**

**Figure S5. Noise Spectral Density of the Sn-Based Perovskite Photodetector Measured at 0 V.**

**Figure S6. Wavelength-dependent detectivity curves are calculated by EQE and noise current under 100Hz.**

**Figure S7. Performance Characterization of the Sn-Based Perovskite Photodetector.** (a) Current density–voltage (*J-V*) curves of the devices measured under dark conditions. (b) Wavelength-dependent detectivity curves were calculated from EQE and dark current at 0 V.

**Figure S8. Performance characterization of the Sn perovskite films.**

Time-resolved photoluminescence decay of Sn-based perovskite films with different methods of passivation.

|  | $\boldsymbol{I=}\boldsymbol{A}_{\boldsymbol{1}}\boldsymbol{e}^{\boldsymbol{-}\frac{\boldsymbol{t}}{\boldsymbol{\tau}_{\boldsymbol{1}}}}\boldsymbol{+}\boldsymbol{A}_{\boldsymbol{2}}\boldsymbol{e}^{\boldsymbol{-}\frac{\boldsymbol{t}}{\boldsymbol{\tau}_{\boldsymbol{2}}}}\boldsymbol{+}\boldsymbol{A}_{\boldsymbol{3}}\boldsymbol{e}^{\boldsymbol{-}\frac{\boldsymbol{t}}{\boldsymbol{\tau}_{\boldsymbol{3}}}}$ | (S4) |
| --- | --- | --- |
|  | $\boldsymbol{\tau}_{\boldsymbol{avg}}\boldsymbol{=}\frac{\boldsymbol{A}_{\boldsymbol{1}}{\boldsymbol{\tau}_{\boldsymbol{1}}}^{\boldsymbol{2}}\boldsymbol{+}\boldsymbol{A}_{\boldsymbol{2}}{\boldsymbol{\tau}_{\boldsymbol{2}}}^{\boldsymbol{2}}\boldsymbol{+}\boldsymbol{A}_{\boldsymbol{3}}{\boldsymbol{\tau}_{\boldsymbol{3}}}^{\boldsymbol{2}}}{\boldsymbol{A}_{\boldsymbol{1}}\boldsymbol{\tau}_{\boldsymbol{1}}\boldsymbol{+}\boldsymbol{A}_{\boldsymbol{2}}\boldsymbol{\tau}_{\boldsymbol{2}}\boldsymbol{+}\boldsymbol{A}_{\boldsymbol{3}}\boldsymbol{\tau}_{\boldsymbol{3}}}$ | (S5) |

Use formula S4 to fit the data and then bring it into formula S5 to find the lifetime.

**Figure S9. Determination of the Relative Dielectric Constant of FASnI₃ Perovskite.** (a) Schematic of the device structures used for measurement. (b) Frequency-dependent capacitance curve for calculating the relative dielectric constant of the perovskite.

|  | $\boldsymbol{C=}\frac{\boldsymbol{\varepsilon}_{\boldsymbol{0}}\boldsymbol{\varepsilon}_{\boldsymbol{r}}\boldsymbol{S}}{\boldsymbol{d}}$ | (S6) |
| --- | --- | --- |

The relative dielectric constant of perovskite was calculated using the equation S6, where $\varepsilon_{0}$is vacuum dielectric constant (8.854$\times$10^−12^ CV^−1^m^−1^), $S$is the area of the device and $d$ is the perovskite film thickness.

**Figure S10. Determination of Defect Densities in FASnI₃ Perovskite.**
(a) Structure of the hole-only devices used for measurement. (b-e) Current density–voltage (*J-V*) characteristics of hole-only devices with different passivation methods.

**Figure S11. Determination of Mobility in FASnI₃ Perovskite.** (a-d) Current density–voltage (*J-V*) characteristics of hole-only devices with various passivation methods.

**Table S1. Comparison of the critical parameters of Sn-Pb perovskite PD.** (Using dark current to calculate detectivity)
